# The joy and pain of being a harm reduction worker: a qualitative study of the meanings about harm reduction in Brazil

**DOI:** 10.1186/s12954-024-00962-7

**Published:** 2024-03-04

**Authors:** João Maurício Gimenes Pedroso, Cristiana Nelise de Paula Araujo, Clarissa Mendonça Corradi-Webster

**Affiliations:** 1 Psychology Department, Faculty of Philosophy, Sciences, and Letters of Ribeirão Preto, University of São Paulo, Ribeirão Preto, SP 14040-900 Brazil; 2 Department of Health Education and Behavior, College of Health and Human Performance, University of Florida, Gainesville, FL 32608 USA; 3 Present Address: Central Paulista University Center – UNICEP, São Carlos, SP 13563-470 Brazil

**Keywords:** Harm reduction work, Qualitative research, Social vulnerability, Substance use, Marginalization

## Abstract

**Background:**

Although harm reduction is highlighted as an effective intervention for alcohol and drug use, a funding gap for harm reduction interventions has been identified, mainly in low- and middle-income countries. In these countries, tensions between abstinence and harm reduction models have impaired the shift from punitive practices to evidence-based interventions committed to guaranteeing the human rights of people who use drugs. Since 2015, the Brazilian government has adopted a more punitive and abstinence-focused drug policy that jeopardizes the care of people who use alcohol and other drugs and the comprehension of the harm reduction workers' perspective in relation to their practice. Therefore, this study aimed to comprehend the meanings constructed by Brazilian harm reduction workers regarding their practices with vulnerable populations amidst a context of political tension.

**Methods:**

We conducted 15 in-depth semi-structured qualitative interviews with harm reduction workers employed in public health services for at least 6 months. Data were analyzed using thematic analysis.

**Results:**

The thematic axis "The joy and pain of being a harm reduction worker in Brazil" was constructed and divided into four major themes: (1) Invisibility of harm reduction work; (2) Black, poor, and people who use drugs: identification with the service users; (3) Between advocacy and profession: harm reduction as a political act; (4) Small achievements matter. Despite the perceived invisibility of harm reduction workers in the public health and alcohol and drug fields, valuing small achievements and advocacy were important resources to deal with political tension and punitive strategies in Brazil. The findings also highlight the important role of harm reduction workers due to their personal characteristics and understanding of drug use behavior, which bring the target audience closer to actions within the public health system.

**Conclusion:**

There is an urgent need to acknowledge harm reduction based on peer support as a professional category that deserves adequate financial support and workplace benefits. Additionally, expanding evidence-based harm reduction interventions and community-based voluntary drug use treatment centers should be prioritized by public policies to address the human rights violations experienced by people who use drugs.

## Background

Harm reduction is a humane and ethical perspective that aims to protect the human rights of people who use alcohol and other drugs (AOD) [1]. This perspective arose in response to the aids pandemic and the extensive history of violation of the most basic rights of people who use drugs across the world [1, 2]. Its core efforts involve promoting social inclusion, guaranteeing their basic rights, disrupting the marginalization of people who use drugs, and decreasing adverse health, social, and economic consequences of drug use to decrease adverse health, social, and economic consequences of drug use without requiring interruption of this use [1, 3]. By helping people who use drugs access resources to become healthier and protect their lives, harm reduction is also protecting their loved ones and communities [4]. Importantly, it has been highlighted as a cost-effective intervention for people who use AOD [5–7].

A literature review study showed that harm reduction in Latin American countries, mainly in Brazil, had been taking a broader outline in the renewal of drug public policies until 2006 [8]. However, since 2016, the harm reduction funding gap has grown mainly in low- and middle-income countries, where funding is only 5% of the required level [6]. Furthermore, extensive literature shows the great threats to human rights of people who use AOD following the growth of long-term residential coercive treatment in Asia [9–11], Australia [10], and Latin America [10–16]. The debate about the lack of commitment of drug policy operators to the promotion of human rights in Latin America has been a topic at United Nations meetings for several years. This debate resulted from the perceived lack of objectivity of the International Drug Control Regime (IDCR) which by combating problems related to the production, trade, and consumption of drugs served as justification for the current mass incarceration of people for activities related to drug trafficking in Latin American countries, such as Bolivia, Ecuador, Peru, and Brazil [17]. The interaction between advocacy coalitions, each composed of actors from various organizations who share a set of beliefs and perceptions of important phenomena, that compete to translate their beliefs into public policies is extensively explored in the Advocacy Coalition Framework (ACF) theory developed by Paul Sabatier and other collaborators [18]. In the AOD field, these coalitions can be characterized by ideological differences related to the care of people who use AOD. The harm reduction coalition believes in a person-centered approach to minimize the harm associated with the use of AOD even when continuing the use. By contrast, the abstinence coalition emphasizes the repression of drug use and treatment programs that aim the total abstinence of AOD.

In Brazil, the first harm reduction experiences were developed in the late 1980s, motivated by the high rates of people infected with HIV [19–21]. Teams of health professionals went to injecting cocaine use scenarios in Santos City to promote health information, prevention interventions, and syringe exchange [19]. This city was also the first to implement a mental health care network that completely replaced the model focused on psychiatric hospitalization in Brazil [22]. These initiatives were great advances for the social movement in favor of quality and dignified care of people in situations of vulnerability [11], contributing to the implementation of harm reduction as a cornerstone of the Brazilian drug policy since 2003 [23].

Yet, since 2015, the Brazilian federal government has strongly reinforced the war on drugs, increasing funding for private long-term residential centers (e.g., psychiatric hospitals and therapeutic communities), underfunding community-based voluntary public services, and undermining the harm reduction paradigm [24–29]. The funding of private long-term residential centers has occurred despite the numerous denouncements of human rights violations within them by the national media and health professionals [30–32]. In 2019, a new Brazilian mental health and drug policy was implemented, emphasizing the long-term residential treatment of children and adolescents, electroconvulsive therapy, and coerced treatment [33]. In 2021, the Global Drug Policy Index showed that Brazil was the lowest-ranking country regarding drug policy compared to 29 other countries [11]. This index compared the degree of implementation of national-level drug policies, considering principles of health, human rights, and development. This period coincided with a historical moment in the country in which the elected federal government was explicitly against harm reduction and in favor of intensifying repressive and punitive approaches to combating drug trafficking. The presence of politicians in power, identified with far-right groups that flirt with violence and intolerance, is an appearance that also arrived in Brazil [34].

Such contradictions have jeopardized the care of people who use AOD and the understanding of the harm reduction workers' perspectives on their practice. This knowledge is crucial for planning evidence-based harm reduction approaches. Although some studies investigated the implications of harm reduction practices for drug use, few explored the experiences of harm reduction workers, especially those formally employed in the Brazilian public services [35]. Herein, this study aims to comprehend the meanings constructed by Brazilian harm reduction workers regarding their practices with vulnerable populations amidst a context of political tension.

## Methods

This is a qualitative and descriptive study. We conducted semi-structured interviews with harm reduction workers from six Brazilian states between September 2018 and August 2019. The interview script was developed through discussion with research staff and health professionals. The questions were created to promote conversations about harm reduction workers' memories, experiences, and practices with the intersectoral health network, their teams, and the people who use AOD.

### Participants and procedures

**Fig. 1:**
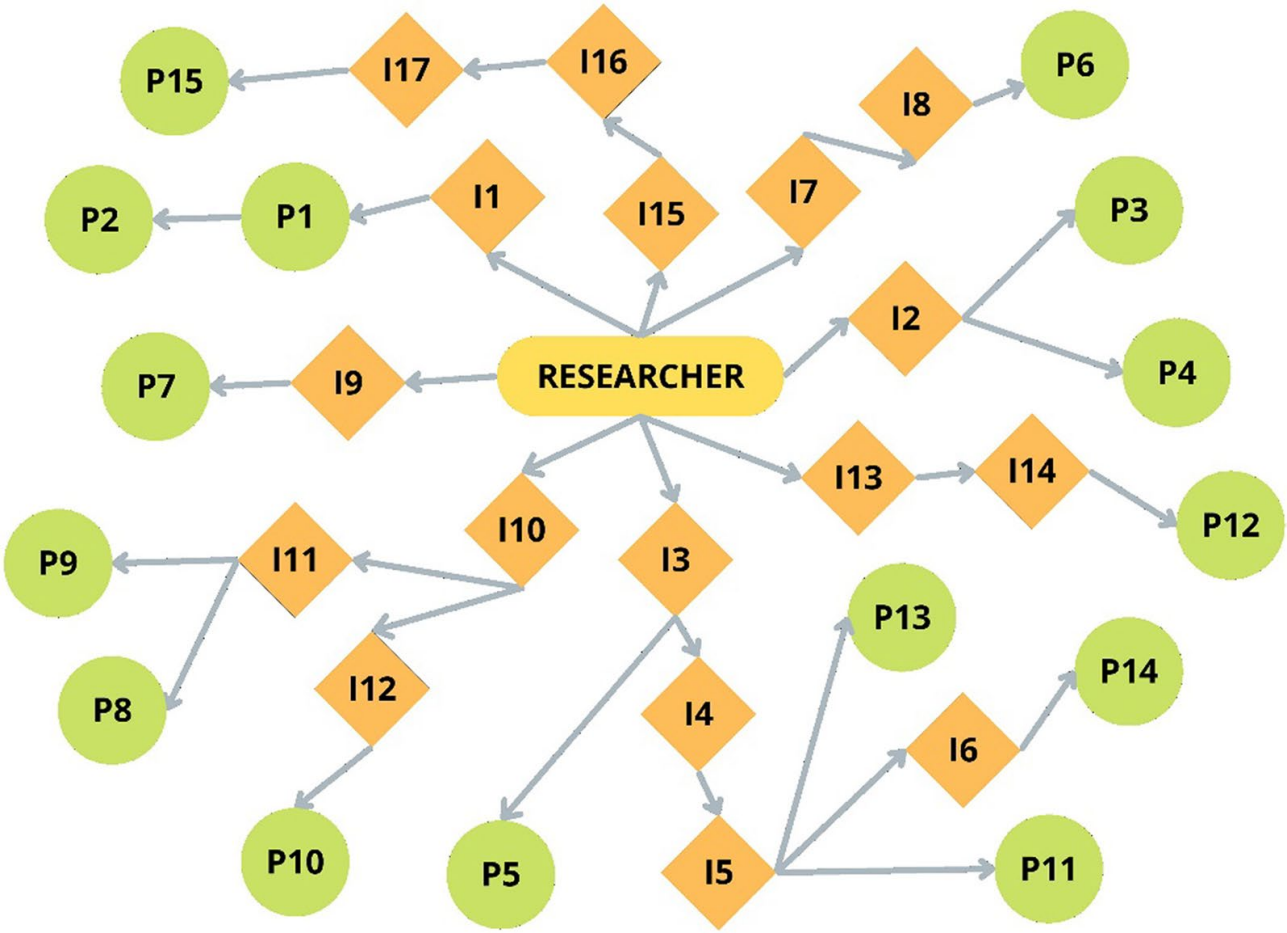
Participant recruitment process through the snowball technique. *Notes* I: informants asked to recommend possible participants; P: participants included

The study sample included 15 harm reduction workers from the public health system, including primary care services, street outreach programs for unhoused people, medium-complexity services related to the HIV policy, community-based voluntary drug use treatment centers, and transitional housing. Because this is an unusual professional category in the Brazilian context, participants were recruited using the snowball sampling technique [36]. The first author asked people from his network and research team (informants)—public health professionals, university professors, and members of non-governmental organizations—to indicate possible participants for the study (Fig. 1). Five people did not meet the inclusion criteria as they were no longer employed as harm reduction workers. However, there was no refusal to participate in the study.

Inclusion criteria were: (1) being employed as a harm reduction worker for at least six months; and (2) working with a vulnerable population. Most participants were women (*n* = 9), with a mean age of 40.45 years (± 10.3). The person with the least experience had been working for 2 years, and the most experienced person for 28 years. All participants reported performing outreach work, predominantly within the Southeast region of the country (*n* = 11) (see Table 1).

**Table 1:** Characteristics of participants

Fictitious name	Gender	Race	State	Age	Education level	Job types	Service	Years working with harm reduction
Paulo	Male	Black	Rio de Janeiro	45	High school	Harm reduction worker	Community-based drug use treatment center	6
Sandra	Female	Black	Rio de Janeiro	38	High school—Undergraduate in Psychology	Harm reduction worker	Community-based drug use treatment center	2
Marcelo	Male	Black	Bahia	54	College—Philosophy with specialization in alcohol and other drug use	Training field supervisor of harm reduction workers	Extension program of a federal university	28
Tania	Female	Black	Bahia	36	High school	Harm reduction worker	Social Program of the Justice and Human Rights, Department of the State of Bahia	17
Danilo	Male	Black	Goiás	42	High school	Social educator	Transitional housing—youth	8
Roberto	Male	Black	Alagoas	50	College—Theatre and accounting	Social educator	Harm reduction street outreach program	10
Claudia	Female	Black	Minas Gerais	63	Incomplete high school	Harm reduction worker	Community-based drug use treatment center	8
Daniela	Female	White	Minas Gerais	50	High school	Harm reduction worker	Association of sex workers	20
Carla	Female	Black	São Paulo	38	High school	Harm reduction worker	Transitional housing—adults	8
Sérgio	Male	White	São Paulo	30	Incomplete college—Social work	Harm reduction worker	Community-based drug use treatment center	7
Cristiane	Female	Asian	São Paulo	32	College—International affairs and economics	Harm reduction and prevention worker	Harm reduction program	2
Bruna	Female	White	São Paulo	29	College—Psychology with specialization in mental health	Harm reduction worker	Harm reduction street outreach program	7
Cleber	Male	Black	São Paulo	28	College—Social science	Harm reduction worker	Community-based drug use treatment center	3
Renata	Female	White	São Paulo	27	Incomplete college—Social science	Social educator	Community-based drug use treatment center	2 and 3 months
Beatriz	Female	Black	São Paulo	45	High school	Harm reduction and health worker	Sexually transmitted infections (STIs), HIV, and viral hepatitis program	5

Individual interviews were conducted in person or remotely via telephone, enabling the inclusion of professionals from other states. The researcher chose a private silent space for the remote interviews to facilitate the audio recording. The face-to-face interviews were carried out in private spaces indicated by the participants. The interviews lasted an average of 47 min, ranging from 15 min to approximately two hours. Interviews lasted approximately one hour and were recorded for later analysis. Participants were interviewed by a clinical psychologist trained in conducting interviews (Author1) after they signed the informed consent form. In cases where the interview took place over the phone, participants printed and signed the informed consent form received via email, then they sent a scanned copy of the signed form via email to Author1. This study was approved by the University of São Paulo Ethics Board (Reference number 88094418.1.0000.5407). Furthermore, there were no reports of discomfort or embarrassment related to the content or format of the interviews.

Data collection concluded upon reaching theoretical data saturation, when no new insights emerged from the data and the complexity of the data was enough to understand the phenomenon studied in depth [37]. Transcription and initial analysis of the data were carried out after the first interview to observe initial codes and the need to explore important emerging issues more deeply. During the data collection, the authors (Author1, Author3) met regularly to read the interview transcripts and discuss initial data analysis, future interviews, and data saturation.

### Data analysis

Data analysis was carried out using the thematic analysis method described by Braun and Clarke [38]. This approach has been widely used by qualitative researchers of numerous disciplines enabled by its flexibility in terms of theoretical framework, research question, sample size and constitution, data collection procedures, and approaches to meaning generation [39]. The purpose of thematic analysis is to search for patterns across the dataset, interpret the data, and discuss the findings considering the literature of the study field, which involves robust, thorough, and systematic processes for coding qualitative data to develop themes that will answer your research question [40, 41]. The results are based on the interpretation of the data in dialogue with the scientific evidence already reported in the literature. The first author transcribed all recorded audio interviews and performed exhaustive readings of the data, noting impressions and reflections for familiarization with the data. Data coding was performed by creating a list of codes and interview excerpts relevant to each code. Codes capture the semantic and conceptual representation of the data. The data and codes were read again, seeking coherent meaning patterns of data considered relevant to constructing the preliminary themes. The themes were reviewed by observing their coherence with their codes and data. Then, the themes were defined and named, and interview excerpts illustrating each theme were selected. The authors (Author1, Author3) developed the data coding, theme generation, theme review, and theme definition processes. As recommended by the authors [42] the initial codes and themes were presented to a group of researchers and professionals with experience in harm reduction, helping to broaden the analysis process and reach the final codes and themes. The data analysis was finalized with the writing of the results by all authors. Participants' quotes selected to illustrate the results were translated by the authors.

## Results

**Fig. 2:**
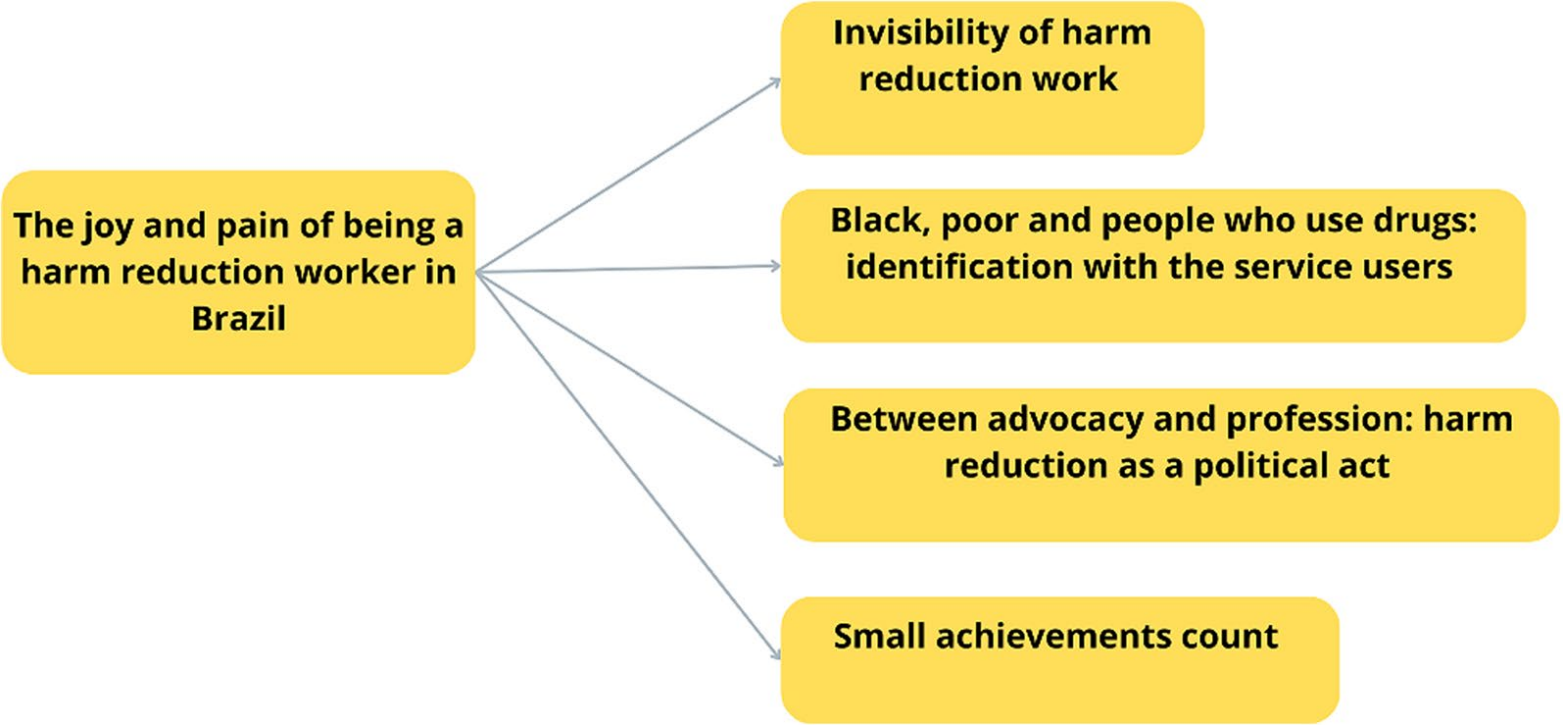
Thematic map of the axis joy and pain of being a harm reduction worker in Brazil

Thematic analysis generated a thematic axis, "The joy and pain of being a harm reduction worker in Brazil", which is divided into four major themes (see Fig. 2).

### Invisibility of harm reduction work

The participants addressed the feeling of devaluation. In general, they perceived that the daily work was impacted by the dismantling of services, as well as the lack of professional training and understanding of the harm reduction workers' role within their team. The perception that their technical knowledge was not validated by other health professionals, the decrease in public funding for harm reduction, and the lack of professional stability contributed to the feeling of devaluation and depreciation of the worker.

Tania noticed that harm reduction workers in Brazil were often assigned tasks not outlined in their job description, such as reception, screening interviews, and bureaucratic activities. She perceived this as a devaluation of harm reduction work, contributing to the loss of their identity as they became responsible for fulfilling tasks unrelated to harm reduction practices.

The harm reduction worker was overloaded with different tasks. He kind of worked in the field, but at the same time, he took care of reception, attendance, and did a little bit of everything, unfortunately. I won't mention the institutions, but some have this kind of attitude, and it's complicated. So, the devaluation of the professional category is a great difficulty for me.

Tania also reported the importance of formally recognizing harm reduction as a profession in order to increase its recognition and pay equity:

So, in general, I think harm reduction has this issue because it is not a formal profession. There are states in Brazil where harm reduction is already a professional category, regulated, and everything is fine... So, I think this is a great pain for harm reduction workers because we are workers and we would like to have the recognition of the profession and the importance of this profession.”

Renata said that she realized that the other professionals of the community-based drug use treatment center devalued the actions of harm reduction workers, treating them in a derogatory way: *"I think that within the community-based drug use treatment center, we are still seen a lot like this: Oh, they are crazy, look at these harm reduction people, it's all one big party…".*

Bruna said there was a lack of courses, supervision, and qualifications for harm reduction workers to improve their practices: *"I think more investment… more educational investment… courses, opportunities to attend lectures, supervision, cultural activities, buying instruments, or better qualifying the team, this would bring a much more effective return."*

### Black, poor, and people who use drugs: identification with the service users

For the participants, the different ways of understanding drug use and situations of vulnerability affect the treatment outcomes and quality. It also influenced the person-focused practice and the willingness to work with people in vulnerable situations. This understanding was constructed through their knowledge of the street culture, the language used by people who use drugs, and their identification with the service users, mostly black and poor. The participants also reported that their work experience enabled their immersion in the street culture, learning how people who use drugs interact and the language used in this context. This immersion was emphasized as a central role of this professional category, allowing a closer approach to users and a better response to their particular needs. Peer work was also highlighted as a strategy based on approximation and building trust between the professional and service users.

Danilo reported that he started smoking marijuana when he was older and this shaped his understanding of drug use as just another way of dealing with the world. He also said the self-identification of harm reduction workers with the service users, particularly in terms of the shared experience of being "black and poor", was the most important resource in their work:

(…) my use is very recent since I was twenty years old. I never had a history of marginalization, but I always saw myself as a poor and black person. So, I experienced many difficulties because of this condition. This is just another way of facing the world. It's another way of positively and actively putting myself in the world and fighting.

As a harm reduction worker in a transitional home, Carla made efforts to carry out activities with the residents outside the service, such as going for walks and participating in workshops offered by the social welfare network. In these situations, she perceived the stigmatization of people who use drugs as an important challenge to the social reintegration of the service users. Therefore, activities in external and public spaces, without identifying the health service of origin, were a strategy to promote social reintegration of the service users and cope with the prejudice.

Working with social welfare services and cultural projects is my initiative. I talk with people, and we articulate this partnership. Not that there is this conversation, we make an exchange. They need people to participate in their workshops. We bring the participants, but it's difficult, it's not easy... Because when you say you're going to bring drug users to a place, people turn up their noses.

Cristiane said that she identified herself as a person who uses drugs, and this facilitated the approach and communication with the target population. She also highlighted that peer work strengthens the alliance between the service users and professionals:

This harm reduction outreach program aims to promote work between peers; that is, you must have something in common with the population with whom you will work. So, we have ex-convicts, black people, and transgender people on the team. In my case, I'm there because I like drugs, to have a conversation more among peers. The bond...

Claudia recognized the importance of knowledge about the street context for properly monitoring people who use drugs. She shared an experience when she accompanied a service user to his medical appointment and helped mediate the communication between him and the doctor, translating the slang used by the user:

I learned the street talk; I'm already good at their slang. Even the doctors do not understand what they say, but we translate. We sometimes ask the patient: "Please translate this language for us". Then they go ahead and explain.

### Between advocacy and profession: harm reduction as a political act

Considering the self-identification and proximity with people who use AOD described by the harm reduction workers, the perceived advances in treatment and improvement in quality of life of people assisted were strongly valued by the participants, while observing the devaluation of their professional category. Therefore, their personal satisfaction obtained from the appreciation of the positive outcomes achieved through affective relationships with the service users was the only reward for their work, which helped them to endure the lack of professional recognition. Furthermore, activism was described as a facet of their work, while financial motivation appeared in the background since it was inadequate. In this way, the work was characterized as part of the harm reduction workers' identity and a source of satisfaction.

Cristiane noted differences in her practice when comparing her experiences as a harm reduction worker in a non-governmental organization and a public health service. She said the non-governmental organization refused to provide care for people under the influence of substances and showed discomfort in making the care offer conditional upon abstinence. She described the importance of low-threshold treatment, with goals tailored to people’s needs without any restrictive criteria limiting care access.

This year I did an internship at another harm reduction institution, a non-governmental organization, where they do harm reduction with people in vulnerable situations, but a little less vulnerable. So generally, they are all living in a shelter. It's more a matter of alcohol than crack. When the person is intoxicated, they don't let them enter the non-governmental organization. So, I realized that when there is this filter, I don't like it. I like working on the street with people who are really crazy.

Roberto shared his history of activism and how working with unhoused people contributed to the construction of his identity. He started working for financial reasons and then fell in love with the task. He emphasized the importance of affection in his work and associated it with person-centered care. He reported being pleased to provide care in a street context because of the great potential of this work:

I have an activism background, and working with unhoused people touched me a lot in the sense of being a human being and of humanization. There are some cases where I have such great affection for the users, as in the case of many elderly ladies I work with who are difficult to work with… to reach. I came to the harm reduction street outreach program due to the salary, and then I fell in love with work due to its enormous power... (...)

For Marcelo, while health professionals are mainly motivated by the financial return for their work, harm reduction workers are motivated by the opportunity to offer respectful care to people who use drugs:

I see that what makes health professionals work is the financial aspect, the salary. The harm reduction workers go beyond that. In addition to earning that money that it is obvious that every professional likes and needs, they offer completely different care because they work on a different logic of seeing people more respectfully.

### Small achievements matter

The participants emphasized the importance of focusing on small achievements to persist in their career amidst the lack of public funding for harm reduction practice and recognition of harm reduction based on peer support as a professional category with adequate financial support. The participants highlighted their satisfaction with the small changes in people's lives, such as establishing therapeutic alliances and trusting relationships with people living in vulnerable situations, improving their living conditions, increasing their self-care behaviors, and helping them re-establish affective bonds with friends and family.

Bruna spoke about the improvement in the general health condition of an unhoused person who was more aggressive and threatened the health team at the beginning of the intervention. Over time, the harm reduction approach helped to develop a friendly and trusting relationship between the team and this person and improved the general health of this person, who was no longer using drugs so harmfully:

She put herself at risk a lot, and sometimes she was bleeding. She threatened the team. She wanted to contaminate us [with HIV]. So, it was very difficult. But today we have a very strong bond with her... And she is fine today. She's using drugs, but it's not as harmful as before.

Tania reported that the indicators of improvement in people's lives should be evaluated according to the singularities of each person because changes did not happen overnight, and they were usually small. She considered being able to communicate and establish a trusting relationship to be noticeable changes:*"… For example, if a person who didn't take shower begins to do so, that's a step. If a person who didn't want to talk or couldn't express herself/himself or was distrustful starts talking, that's harm reduction".*

Renata cited an example of how the harm reduction approach promotes rehabilitation by improving the organization of people's lives, financial issues, and housing: *“We observed, through our conversations, that the woman had organized herself and her life. She started working in a social and solidarity cooperative program, renting a room in a pension”.*

In this way, the harm reduction workers demonstrated the impact of empathy on their motivation to perform the work. The treatment outcomes observed, including the reorganization process, improvement in autonomy, and the establishment of trusting relationships with the people who use AOD, represented a professional reward considering the context of political tension.

## Discussion

Although these findings focus on harm reduction in Brazil, they have implications for a broader understanding of these practices. The funding gap for harm reduction and poor mapping of harm reduction actions are increasing in low- and middle-income countries [6, 43]. This gap is accompanied by the implementation of actions characterized by extreme human rights violations, mainly in low- and middle-income countries, such as extrajudicial executions (widespread in Mexico and endemic in Brazil), militarized drug law enforcement (endemic in Brazil, Colombia, Kyrgyzstan, Mexico, and North Macedonia), life imprisonment for drug-related offenses, and the involuntary confinement of people who use drugs (widespread in Afghanistan, Brazil, Thailand, and Uganda, and endemic in Mexico) [11].

Participants highlighted the invisibility of harm reduction workers associated with the disinvestment in harm reduction by the Brazilian government, and the lack of professional training, understanding of their role among other health professionals, and professional stability. Similar results were observed by Greer et al. [44, 45], who showed that peer work in the AOD field in Canada was precarious, characterized by instability and job insecurity with limited wages and social benefits. Precarious work relationships are exacerbated by chronic underfunding [46] and delegitimization of the drug user rights movement by national policymakers [43]. Some policymakers may be reluctant to invest in developing formal harm reduction territorial policies and may prefer to focus on other more pressing concerns, including the worsening overdose epidemic and other potentially more expedient strategies to secure resources [47]. Despite the strong need for the harm reduction strategy and scientific evidence supporting its benefits for people who use drugs, the abstinence coalition will resist arguments suggesting its core beliefs may be invalid and/or unattainable, and use formal political analysis to strengthen its beliefs and/or attack their opponents [18].

In some Brazilian municipalities, the support of powerful policymakers by largely religious private-sector long-term residential centers may be associated with the significant increase in these institutions while undermining community-based voluntary drug use treatment and harm reduction approaches [48]. In Brazil, the underfunding of policies and services guided by the harm reduction perspective is a phenomenon directly related to the strengthening of long-term residential centers [49] despite the numerous reports of human rights violations within these institutions [30–32]. These changes in the drug policy can be better understood by analyzing the political tension between the abstinence coalition, composed of actors linked to long-term residential institutions, and the harm reduction coalition, composed of actors who reclaim the financing of outpatient care provided by public services [18]. The abstinence coalition may be more able to raise budgets, recruit more supporters, and legal authority influencing veto players’ decisions regarding a drug policy focused on abstinence that favors specific groups of the abstinence coalition [18]. The war on drugs restricts evidence-based health strategies, such as harm reduction, and generates the proliferation of punitive approaches based on abstinence and involuntary confinement in low- and middle-income countries [9, 12–16, 24, 28, 29, 50, 51]. Certainly, tensions between abstinence-based treatment and harm reduction approaches represent a major challenge for a shift in the drug policy paradigm in countries that have heavily adopted coercive and punitive interventions [48, 52, 53].

This polarization between abstinence and harm reduction models in the context of public drug policy impacts harm reduction workers, causing their invisibility. However, most participants self-identified with the people enrolled in treatment as they reported being black, poor, and people who use drugs, experiencing the invisibility and inequities also lived by the service users. This shared experience motivated them to advocate for expanding health care based on human rights for people who use drugs. Accordingly, harm reduction interventions based on peer support have a key role in building a therapeutic alliance with people who use drugs [54, 55], promoting treatment engagement [56–58], and providing quality care for minority groups [59, 60], as harm reduction workers have lived experiences similar to the service users and high levels of empathy. This approach is fundamental since [61] people who use AOD experience a high level of stigma [62, 63] and, as a result, have lower access to treatment in low- and middle-income countries [50, 54, 57, 64]. A study conducted in 20 countries showed that people from countries with prohibitionist drug policies had lower help-seeking rates compared to countries with relatively liberal drug policy regimes [65]. Furthermore, this population is disproportionately targeted by drug law enforcement and faces discrimination throughout the criminal system [1, 66]. Brazil has adopted a more repressive drug policy, in which ethnic, low-income, and gender groups perceived more obstacles in accessing harm reduction [11], with the Brazilian black community being the most affected [67]. Similar to the history of social struggles experienced by the black community in search of recognition and dignity in the USA, racism is a determining issue for the impacts of drug policy on black communities and the promotion of human rights in a multiracial country as Brazil [61]. These minority groups are the main actors involved in the harm reduction coalition which has more difficulty in maintaining an effective presence in the construction of such policy over time when compared to the abstinence coalition that involves stakeholders of religious private-sector long-term residential centers. Economically disadvantaged groups face greater challenges in gathering the resources needed to remain active over an extended period of time [18]. This imbalance of forces prevents the translation of the harm reduction coalition’s beliefs into public policies.

The results showed the importance of the harm reduction workers' self-identification for the peer-support relationship and their political positioning, with advocacy in favor of harm reduction practices and the provision of quality health care for people who use AOD becoming part of their identity. A study showed that bottom-up grassroots activism led to the spread of harm reduction strategies (often involving approaches not yet discussed by public health agencies) that were central to the subsequent reduction in the impact of HIV in New York City, Rotterdam, Buenos Aires, and sites in Central Asia [2]. The consolidation of harm reduction as a global movement was strongly influenced by the self-organization of groups of people who used drugs affected by the aids pandemic in the 1980s across the world [2].

In Brazil, the fight for public health care access and the strengthening of harm reduction approaches also occurred simultaneously. The first needle exchange experiences were carried out unofficially by groups of people with lived experience of drug use, professionals, and managers who took risks and trusted in this care model that transcended the legal boundaries since it was based on historical fights of social movements [19]. Therefore, despite the disinvestment in public health policies that aim to guarantee universal health access, the participation of Brazilian society is important for the continuity of harm reduction [68, 69]. Harm reduction theory and practice should consider micro-social activities and formally organized groups of people who use drugs [2, 70]. The most effective harm reduction approaches with lasting effects have integrated both bottom-up grassroots activism and top-down public health approaches to respond to the needs of people who use drugs [71].

In this way, the participation of different actors of civil society in the dispute for the promotion of changes in public policies is decisive, especially at a time of advancement of far-right governments. The political instability that the country is going through, added to the various players with veto power who occupy decision-making spaces in the Congress and Senate, make it difficult to make lasting changes toward strengthening policies aimed at promoting human rights for people who use drugs. Despite the limited decision-making spaces, it is clear that the harm reduction coalition must keep political decisions in constant discussion and debate, acting as veto players in the political game [72]. To enable this, it is necessary to find ways to bring together, through advocacy coalition, stakeholders from multiple positions (elected interest group leaders and agency officials, harm reduction activists, groups of people who use drugs, community-based organizations, health professionals, and researchers) who share a set of basic values, assumptions, causal factors, and problem perceptions [18]. This role proves to be decisive for maintaining the advances achieved with the harm reduction social movement.

The participants’ activist stance led to the appreciation of small achievements of the people in care, helping them endure the devaluation of their practices, lack of public funding, and nonrecognition of harm reduction based on peer support as a professional category. These small achievements were highlighted as a reason for their professional satisfaction and to continue working actively to consolidate harm reduction in their communities. Similarly, the desire to improve the lives of people in vulnerable situations and to learn from their experiences were described as motivational factors by other Brazilian harm reduction workers [73]. The strong commitment of harm reduction workers is critical in ensuring effective care for people who use drugs, mediating the relationship with the health service [54, 55]. Peer support facilitates treatment engagement [56–58] and is widely used to reduce the transmission of sexually transmitted infections among vulnerable groups [59]. The integration of housing first, harm reduction, and peer support demonstrated effectiveness in decreasing substance use and improving the quality of life of unhoused people over time [60]. Successful harm reduction programs actively seek out the people and are flexible regarding their treatment goals and needs [54, 74], creating a low-demand context that promotes their autonomy [75]. A systematic literature review showed that unhoused people using AOD preferred harm reduction-based treatments as they provided person-centered care, a facilitative environment, empathic and non-judgmental support, and opportunities to relearn how to live [76]. This person-centered care expands the horizons of care and professional performance and guarantees comprehensive care [77]. Accordingly, peer support plays an important role in harm reduction practices, and efforts are needed to ensure that peer workers receive adequate financial support and workplace benefits [57].

### Limitations

Our findings may be circumscribed by the concentration of participants in the southeastern region of Brazil and the heterogeneity of the workplaces of each participant. The results do not allow generalization of the meanings constructed by the participants regarding harm reduction to all of Brazil. However, they provide an overview of these practices in the public health context through dialogue with different actors amidst setbacks in drug and social policies. Not having included the perspective of service users can also limit the meanings constructed. However, the focus on the narratives of actors who are historically protagonists in the construction of harm reduction interventions may allow a deeper understanding of the subject. Future studies should examine the effectiveness and long-term impact of harm reduction approaches for unhoused people. Another limitation includes conducting the thematic analysis without using any software which may increase the rigor of the analysis. However, the size of the sample allowed a systematic and thorough engagement with the data without software while keeping an efficient organization of the coding. Additionally, two authors participated in the data coding, theme generation, theme review, and theme definition processes to ensure that the themes generated were meaningful within the aim of the current study. Furthermore, the sample of participants consisted of people identified as cisgender, which may have limited pertinent analyses within the LGBTQIA+ community, a community historically oppressed and violated by prohibitionist drug policies.

## Conclusions

This study highlights important insights about the meanings constructed by Brazilian harm reduction workers regarding their practices with vulnerable populations amidst a context of political tension. The participants described their invisibility in the public health field as reflected by the precarious work conditions and the delegitimation of their practices. This was shaped by the inequities experienced by them and the population enrolled in treatment, as they were mainly black, poor, and people who use drugs. Only those harm reduction workers who embrace activism and value even the smallest achievements conquered by people who use drugs can endure this rough reality and continue working to strengthen harm reduction practices. Ongoing barriers to implementing the harm reduction model include the historical tension between the abstinence and harm reduction approaches and the Brazilian government's repressive agenda based on abstinence and the war on drugs. The importance of the harm reduction workers' role was also highlighted due to their personal characteristics and understanding of problematic drug use to construct a therapeutic relationship and increase access to health care among hard-to-reach groups. These findings reveal the need for international pressure for the paradigm shift from punitive practices based on abstinence to harm reduction aiming to reduce the inequalities in treatment access in Brazil. It is important to consider the role of workers, users, researchers, and organizations in rebuilding harm reduction policies after a period of setbacks in the promotion of evidence-based drug policies. This is a field in constant dispute, influenced by lobbies and political financing, public opinion campaigns, and the construction of alliances as strategies for the implementation or veto of certain public policies that might favor the elites rather than voters. The recognition of harm reduction based on peer support as a profession with adequate financial support and workplace benefits, and a public policy committed to expanding evidence-based voluntary community-based drug use treatment are urgently needed to address human rights violations of people who use drugs.

## Data Availability

The datasets analyzed during the current study are not publicly available due to confidentiality to preserve the anonymity of participants but are available from the corresponding author on reasonable request.

## Abbreviations

AOD: Alcohol and other drugs
HIV: Human immunodeficiency virus
